# Diastolic Dysfunction with Normal Ejection Fraction and Reduced Heart Rate in Mice Expressing Human Growth Hormone and Displaying Signs of Growth Hormone Insufficiency

**DOI:** 10.3390/ijms26010269

**Published:** 2024-12-31

**Authors:** Yan Jin, Bo Xiang, Vernon W. Dolinsky, Elissavet Kardami, Peter A. Cattini

**Affiliations:** 1 Department of Physiology and Pathophysiology, Rady Faculty of Health Sciences, University of Manitoba, Winnipeg, MB R3E 0J9, Canada; yan.jin@umanitoba.ca; 2 Department of Pharmacology and Therapeutics, Rady Faculty of Health Sciences, University of Manitoba, Winnipeg, MB R3E 0J9, Canada; 3 Human Anatomy and Cell Science, Rady Faculty of Health Sciences, University of Manitoba, Winnipeg, MB R3E 0J9, Canada

**Keywords:** hGH, transgenic mice, GH deficiency, heart, echocardiography, high-fat diet

## Abstract

Growth hormone (GH) signaling is essential for heart development. Both GH deficiency and excess raise cardiovascular risk. Human (h) and mouse (m) GH differ structurally and functionally: hGH binds both the GH receptor (GHR) and prolactin receptor (PRLR), whereas mGH binds only GHR; thus, there is the potential for differential effects. We generated transgenic (hGH-TG) mice that produce pituitary hGH in response to hypothalamic signaling. These mice grow at the same rate as mGH-expressing wild-type (mGH-WT) mice but are smaller and have higher body fat. Echocardiography was used here to compare hGH-TG and mGH-WT mouse hearts. Male hGH-TG mice show a 48% lower left ventricular mass, 36% lower stroke volume, and 48% reduced cardiac output, resembling GH deficiency. Diastolic dysfunction, restrictive ventricular filling, and lower heart rate are suggested in hGH-TG mice. No significant differences in ejection fraction or fractional shortening were observed, even after high-fat diet (HFD) stress. HFD did not affect RNA markers of cardiac damage, although a possible association between B-type natriuretic peptide RNA levels and heart rate was detected. These observations suggest that diastolic dysfunction related to hGH and/or low GH might be offset by a lower heart rate, while structural changes precede functional effects.

## 1. Introduction

Growth hormone (GH) plays an important role in the growth and development of the mammalian heart and helps maintain cardiac structure and function into adulthood [1,2,3]. Many but not all of the actions of GH are due to induction of insulin-like growth factor I (IGF-I) expression as a result of GH receptor binding and activation of the Janus kinase and signaling transducer and activator of transcription (Jak/Stat) pathway in the liver, as well as peripheral tissues including the heart [4]. Although not the only signals, the major pathways activated downstream in the cardiovascular system include the extracellular signal-regulated kinase (ERK)/mitogen-activated protein kinase (MAPK) and phosphatidylinositol 3-kinase (PI3K)/serine/threonine kinase or protein kinase B (Akt) cascades [5]. The GH/IGF-I axis not only promotes growth and metabolic effects but also influences contractility and the vascular system [4]. More specifically, IGF-I in the cardiovascular system is linked to vasoconstriction/vasodilation, cardiac apoptosis and autophagy, inflammatory-related responses, and promoting angiogenesis [5]. Both an excess and deficiency in GH and/or IGF-I are linked to cardiac dysfunction and an increased risk of cardiovascular mortality [3,6]. Excess GH/IGF-I is often associated with concentric cardiac hypertrophy and diastolic dysfunction, which will lead to systolic dysfunction and heart failure if GH/IGF-I levels are not controlled [6]. This can be exacerbated by the presence of other cardiovascular risk factors related to GH levels, including insulin resistance, dyslipidemia, and hypertension [6]. Similarly, whether in childhood or later, GH insufficiency together with these cardiovascular risk factors can lead to adverse effects on cardiac structure and systolic function [6]. Furthermore, short-term treatment with GH has been proposed as beneficial in the treatment of cardiovascular diseases [7]. Thus, both an excess of GH with acromegaly and GH deficiency (GHD) are associated with an increased risk for cardiovascular morbidity and mortality [6].

While the importance of GH/IGF-I signaling for development and maintenance of the heart is supported by the adverse cardiovascular effects observed in GH receptor (GHR) null mice, normal cardiac function was seen in transgenic mice where the GHR was disrupted specifically in the heart [8]. Whether these findings are applicable to humans is uncertain, since primate and non-primate GH genes differ significantly in structure, and thus human GH (hGH) and mouse (mGH) have the capacity for different regulation, receptor binding, activities, and functions [9,10]. As a result, hGH, but not mGH, can bind the prolactin (PRL) receptor (PRLR) with high affinity in addition to GHR [9,11]. This may have implications since PRL as well as GH receptors are present in the rodent heart and cardiac myocytes [11,12,13]. While it remains unclear whether changes in PRL levels affect the risk of cardiovascular events [14], chronic exposure to high or low PRL levels has been associated with a number of conditions, including atherosclerosis, dyslipidemia, endothelial dysfunction, hypertension, increased visceral fat, and insulin resistance, that are expected to increase the risk of cardiovascular disease [14,15].

To examine potentially different effects of hGH and mGH, we generated transgenic (TG) mice on an outbred CD-1 genetic background that express hGH, but not mGH, specifically in the pituitary and with the ability to respond to hypothalamic control [16]. Use of outbred mice is preferable for biomedical research as they may better reflect genetic variability and applicability to humans [17,18]. These hGH-TG mice were compared with mGH-expressing wild-type (WT) CD-1 mice. Mouse size, bone density, total body fat, glucose clearance, and insulin production in hGH-TG and mGH-WT mice were compared at 28 weeks, as well as the effect of high-fat diet (HFD) for 24 weeks versus regular chow diet (RCD) [16]. The hGH-TG mice displayed signs of some GH insufficiency based on relative size and body fat as well as evidence of prolonged insulin resistance beyond the transient period associated with puberty when maintained on a HFD [16].

Here we have extended this study by assessing heart structure and function in 28-week-old male hGH-TG mice by echocardiography. In addition, we investigated the effect of diet-related stress on hGH-TG mouse hearts by echocardiography and also determined levels of messenger RNA markers linked to cardiac damage. These include atrial natriuretic peptide (ANP) and B-type natriuretic peptide (BNP) transcripts that are upregulated in the ventricles during hypertrophy [19] and elevated collagen type I (COL I) and collagen type III (COL III) as indicators of fibrosis [20]. As markers of cardiac injury related to high blood pressure, we also measured insulin-like growth factor binding protein 5 (IGFBP5) and ring finger protein 207 (RNF207) levels that were previously identified through whole transcriptome analysis in the mouse [21]. Our findings are discussed in relation to the potential for differential effects of GH levels and hGH compared to mGH on the mouse heart.

## 2. Results

### 2.1. Comparison of Male hGH-TG and mGH-WT CD-1 Mouse Body and Heart Weight

**Figure 1:**
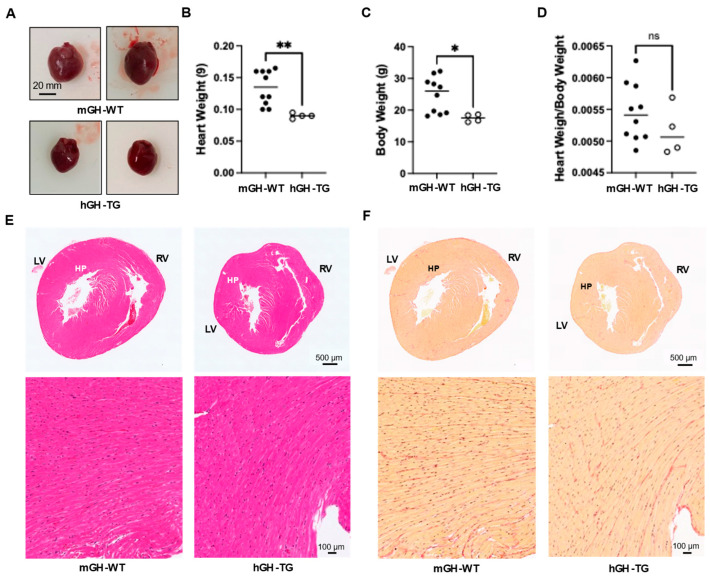
(**A**) Examples of hearts from 4-week-old male mGH-WT and hGH-TG mice (*n* = 4–10). Mean heart weight (**B**), body weight (**C**) and heart weight/body weight ratios (**D**) were determined for 4-week-old male mGH-WT mice (filled circles) and hGH-TG mice (open circles), as well as the ratio for mice at 28 weeks. For each test group, values assessed for each mouse are shown as well as the group mean (horizontal line). Results were analyzed using the Mann–Whitney test (* *p* < 0.05, ** *p* < 0.01, ns = not significant). Adjacent transverse sections through 4-week-old male mGH-WT and hGH-TG mouse hearts, stained with either (**E**) hematoxylin and eosin showing left (LV) and right ventricles (RV) or (**F**) picro-sirius red for interstitial collagen. Regions indicated by HP are shown at a higher magnification in the panel below.

Hearts from 4-week-old male hGH-TG mice were visibly smaller and weighed less than those from age-matched mGH-WT mice (Figure 1A,B), which appears consistent with the smaller mouse size based on length (~20% shorter from head to tail [16]) and body weight (Figure 1C). There was no significant difference between mean heart weight/body weight ratios determined for hGH-TG and mGH-WT mice (Figure 1D). Hearts were also fixed, sectioned transversely, and stained with H&E to compare general ventricular morphology, which appeared proportionate in both mouse types (Figure 1E). Staining with picro-sirius red also suggested similar levels of interstitial collagen deposition, and there was no evidence of significant fibrosis (or fibrous collagen) in both mouse types (Figure 1F).

### 2.2. Comparison of Cardiac Structure and Function in 28-Week-Old Male hGH-TG and mGH-WT CD-1 Mice by Echocardiography

**Figure 2:**
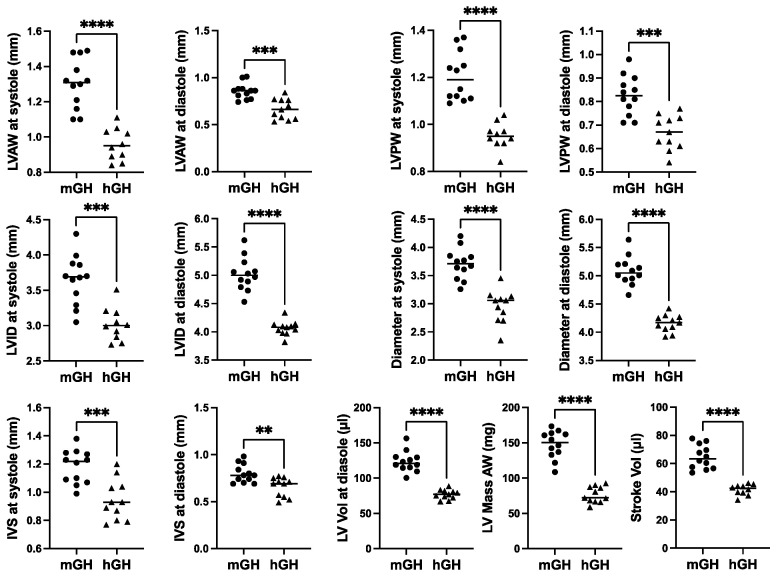
Cardiac structure in 28-week-old male mGH-WT (filled circles, *n* = 12) and hGH-TG (filled triangles, *n* = 11) CD-1 mice maintained on an RCD were assessed using echocardiography, and the results were compared. Assessments included left ventricular (LV) anterior and posterior wall (LVAW and LVPW) thickness at systole and diastole, LV internal diameter (LVID) and diameter at systole and diastole, interventricular septum (IVS) thickness at systole and diastole, LV volume (Vol) at diastole, LV Mass and Stroke Vol. Mean values determined for each mouse type (horizontal line) as well as values assessed for each mouse are shown. Significance was assessed using the Mann–Whitney test and considered significant if *p* < 0.05 (** *p* < 0.01, *** *p* < 0.001 or **** *p* < 0.0001).

Cardiac structure and function in 28-week-old mGH-WT and hGH-TG CD-1 mice maintained on an RCD were assessed using echocardiography, and the results were compared using the Mann–Whitney test. Consistent with the reduced heart weight, multiple cardiac structural parameters, including LV anterior and posterior wall (LVAW and LVPW) thickness and diameter as well as interventricular septum (IVS) thickness, were significantly 17–26% (*p* < 0.01) lower in hGH-TG compared to mGH-WT mouse hearts (Figure 2). In addition, LV mass and stroke volume were significantly 48% and 36% (*p* < 0.0001) lower in hGH-TG compared to mGH-WT CD-1 mice by echocardiography (Figure 2), consistent, at least in part, with the relatively smaller size of hGH-TG mice (Figure 1) [16].

**Figure 3:**
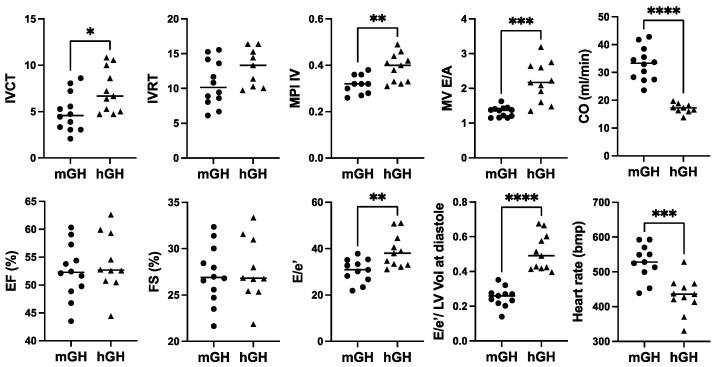
Comparison of cardiac function in 28-week-old male mGH-WT (filled circles, *n* = 12) and hGH-TG (filled triangles, *n* = 11) CD-1 mice fed a regular chow diet by echocardiography. Assessments included isovolumic contraction time (IVCT), isovolumic relaxation time (IVRT), left ventricular (LV) isovolumic myocardial performance index (LV MPI), rate at which the atrial and ventricular pressures equilibrate after onset of the E wave (MV E/A), cardiac output (CO), LV ejection fraction (EF), fractional shortening (FS), mitral inflow E wave to early diastolic mitral annular tissue velocity (E/e′), ratio of E/e′ divided by left ventricular end-diastolic volume (LV Vol-d), and heart rate. Mean value determined for each mouse type (horizontal line) as well as values assessed for each mouse are shown. Significance was assessed using the Mann–Whitney test and considered significant if *p* < 0.05 (* *p* < 0.05, ** *p* < 0.01, *** *p* < 0.001, or **** *p* < 0.0001).

In terms of cardiac function, there was a modest increase in IVCT, a cardiac time interval that, together with IVRT, are components of the myocardial performance index (MPI; sum of IVCT and IVRT divided by ejection time), which was significantly 1.5-fold (*p* < 0.01) higher in hGH-TG compared to mGH-WT mouse hearts (Figure 3), which is indicative of impaired myocardial performance. There was no significant difference between either percent ejection fraction (EF) or fractional shortening (FS) between hGH-TG and mGH-WT mouse hearts (Figure 3). However, the rate at which the atrial and ventricular pressures equilibrate after onset of the E wave (MV E/A) in 28-week-old mice was 1.64-fold higher (*p* < 0.001) in hGH-TG mouse hearts, reaching a mean value of >2.0 (Figure 3). In addition, the E/e′ ratio reflecting mitral inflow E wave to early diastolic mitral annular tissue velocity as well as the E/e′ to LV end-diastolic volume ratio were both elevated significantly in hGH-TG relative to mGH-WT mice (Figure 3). The mean heart rate was also significantly ~18.2% lower (*p* = 0.0004) in hGH-TG mice (~432 bpm) compared to mGH-TG mice (~528 bpm) (Figure 3). Finally, cardiac output (CO), which is calculated by multiplying stroke volume with heart rate, was significantly ~48.3% lower in hGH-TG mice (Figure 3), presumably again reflecting at least in part the smaller relative size of the hGH-TG mouse (Figure 1) [16].

### 2.3. Effect of HFD on the Heart in hGH-TG Mice Showing Signs of Insulin Resistance

**Figure 4:**
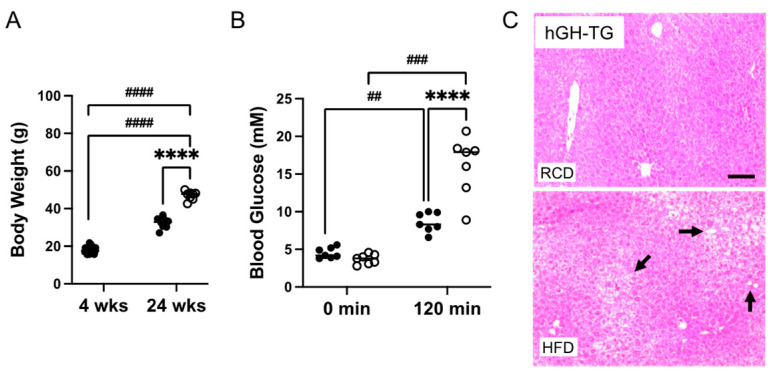
The effect of a (**A**) high-fat diet (HFD) versus maintaining a regular chow diet (RCD) on body weight gain in 4-week-old male hGH-TG CD-1 mice (*n* = 8–24) for 24 weeks was assessed. Blood glucose levels for 28-week-old (**B**) hGH-TG mice measured prior to injection of 2 g/kg glucose (0 min) and 120 min after the injection are shown for mice maintained on RCD (filled circles, *n* = 7) or fed an HFD for 24 weeks (open circles, *n* = 7). Results were analyzed by two-way ANOVA (time, # and diet, *) with Tukey’s *post hoc* test (## *p* < 0.01, ### *p* < 0.001, or ####/**** *p* < 0.0001). For each test group, values assessed for each mouse are shown as well as the group mean (horizontal line). Examples of hematoxylin and eosin-stained liver sections are also shown from 28-week-old (**C**) hGH-TG mice maintained on RCD or fed an HFD for 24 weeks. Lipid droplet features are more evident in livers from mice fed an HFD and examples are indicated with arrows. Bar = 50 μM.

Four-week-old male hGH-TG mice were either maintained on an RCD or fed an HFD for 20–24 weeks, and effects on heart structure and function as well as body weight, glucose clearance, and liver histology were assessed. Data were analyzed using the *t*-test or two-way analysis of variance (ANOVA) where appropriate. Mice fed an HFD weighed significantly ~1.5-fold more than age-matched mice maintained on an RCD at 24 weeks (Figure 4A). A glucose tolerance test (GTT) was also performed prior to termination of the study. There was no significant difference between fasting glucose levels for mice fed HFD versus RCD just prior to glucose injection (time 0 min; Figure 4B). Glucose levels were compared 120 min post glucose injection and were significantly higher than fasting levels regardless of diet. However, glucose levels were significantly 1.9-fold higher (*p* < 0.0001) in mice fed HFD than those maintained on RCD (Figure 4B), which is consistent with greater impaired glucose clearance [14]. In addition, liver tissue from euthanized hGH-TG mice was fixed, sectioned, and stained with H&E at the termination (28 weeks) of the study. The livers of mice fed HFD or RCD were assessed for lipid droplet features as reported by others [20], and representative images from mice fed either HFD or RCD are shown. Increased droplets are visible in livers from mice fed HFD for 24 weeks (Figure 4C), which is consistent with increased lipid accumulation [14].

**Figure 5:**
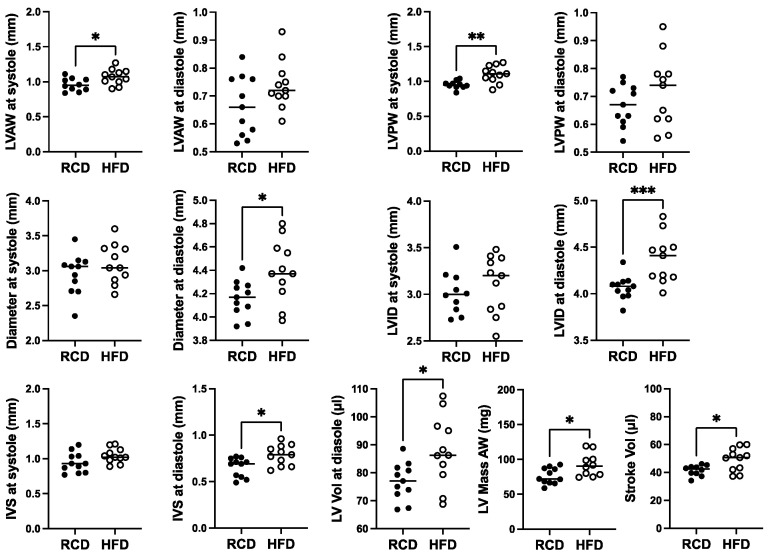
The effect of a high-fat diet (HFD; open circles, *n* = 11) versus regular chow diet (RCD; filled circles, *n* = 11) on cardiac structure in 28-week-old male hGH-TG CD-1 mice by echocardiography. Assessments included left ventricular (LV) anterior and posterior wall (LVAW and LVPW) thickness at systole and diastole, LV internal diameter (LVID) and diameter at systole and diastole, interventricular septum (IVS) thickness at systole and diastole, LV volume (Vol) at diastole, LV Mass and Stroke Vol. Mean value determined for each diet (horizontal line) as well as values assessed for each mouse are shown. Significance was assessed using the Mann–Whitney test and considered significant if *p* < 0.05 (* *p* < 0.05, ** *p* < 0.01, or *** *p* < 0.001).

In terms of effects of chronic HFD for 24 weeks, modest ~10% increases in the diameter and internal diameter as well as LV anterior, posterior, and IVS thicknesses were suggested at diastole (Figure 5). However, only the effect on diameter, internal diameter, and IVS thickness at diastole reached significance (*p* < 0.05). Mild but significant 1.2-fold increases in LV mass and stroke volume were also observed, as well as a 1.1-fold increase in LV anterior and posterior wall thickness at systole (Figure 5).

**Figure 6:**
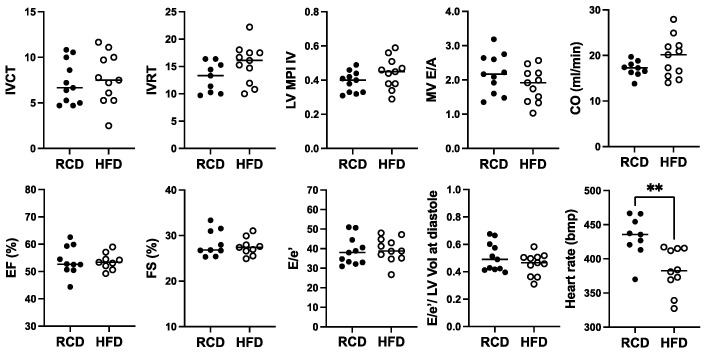
The effect of a high-fat diet (HFD; open circles, *n* = 11) versus regular chow diet (RCD; filled circles, *n* = 11) on cardiac function in 28-week-old male hGH-TG CD-1 mice by echocardiography. Assessments included isovolumic contraction time (IVCT), isovolumic relaxation time (IVRT), left ventricular (LV) isovolumic myocardial performance index (LV MPI), rate at which the atrial and ventricular pressures equilibrate after onset of the E wave (MV E/A), cardiac output (CO), LV ejection fraction (EF), fractional shortening (FS), mitral inflow E wave to early diastolic mitral annular tissue velocity (E/e′), ratio of E/E′ divided by left ventricular end-diastolic volume (LV Vol), and heart rate. Mean value determined for each diet (horizontal line) as well as values assessed for each mouse are shown. Significance was assessed using the Mann–Whitney test and considered significant if *p* < 0.05 (** *p* < 0.01).

For cardiac function, a significant ~11.4% reduction in heart rate from ~432 bpm to ~382 bpm was observed in hGH-TG mice fed with HFD for 24 weeks, but no significant effect of chronic HFD was seen on percent EF or FS, cardiac output, or cardiac time intervals, IVCT, and IVRT, as well as related MPI (Figure 6). Furthermore, there was no significant effect of HFD on MV E/A, E/E′ ratio, or the E/E′ to LV end-diastolic volume ratio (Figure 6).

### 2.4. Effect of Chronic HFD on RNA Levels for Markers Linked to Cardiac Damage

**Figure 7:**
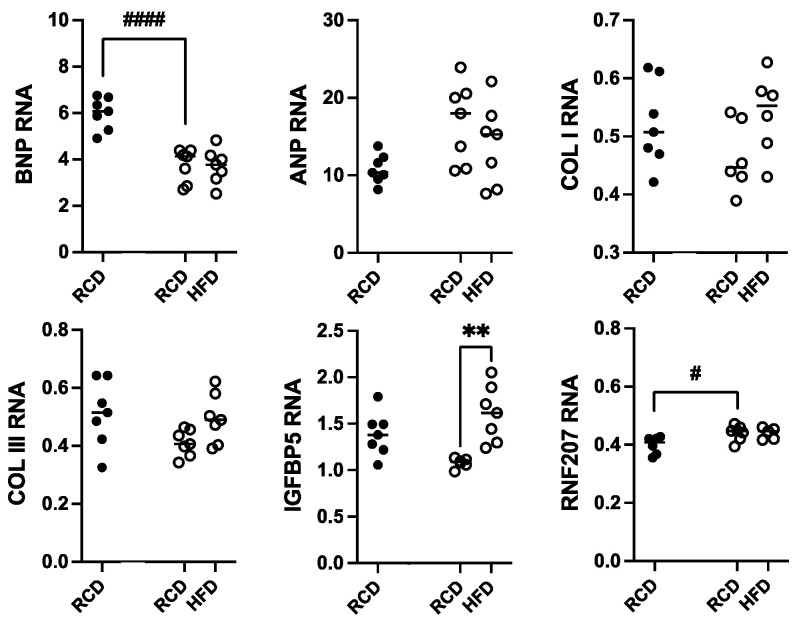
Assessment of heart RNA levels in male hGH-TG CD-1 mice (open circles, *n* = 7) fed high-fat diet (HFD) for 24 weeks versus mice maintained on a regular chow diet (RCD, *n* = 7). RNA from age-matched mGH-WT CD-1 mice (filled circles, *n* = 7) fed RCD were included for comparison. Total RNA was assessed relative to hypoxanthine guanine phosphoribosyltransferase transcripts with specific primers by qPCR for atrial natriuretic peptide (ANP), B-type natriuretic peptide (BNP), collagen type I (COL I) and collagen type III (COL III), insulin-like growth factor binding protein 5 (IGFBP5), and ring finger protein 207 (RNF207). Results were assessed by two-way ANOVA with Tukey’s *post hoc* test. For each test group, values for each mouse are shown as well as the group mean (horizontal line). Significant differences related to mouse type (# *p* < 0.05, #### *p* < 0.0001) and diet (** *p* < 0.01) are indicated.

Total RNA was isolated from heart samples from hGH-TG CD-1 mice maintained on an HFD versus RCD for 24 weeks; RNA from age-matched mGH-WT mice maintained on RCD was included for comparison. Transcript levels for genes linked to cardiac damage, including hypertrophy (ANP and BNP), cardiac fibrosis (COL I and COL III), and hypertension-related injury (IGFBP5 and RNF207), were determined by qPCR. These values were normalized to hypoxanthine guanine phosphoribosyltransferase (HPRT) RNA levels, and each data set was analyzed by two-way ANOVA followed by a Tukey’s *post hoc* test where appropriate (Figure 7). With the exception of a 37.3% lower BNP (*p* < 0.0001) and modest 1.2-fold higher RNF207 (*p* < 0.05) RNA levels in hGH-TG mouse hearts, there were no significant differences in ANP, COL I, COL III, and IGFBP5 transcript levels between mouse types fed RCD at 28 weeks. Furthermore, with the exception of a modest 1.5-fold increase in IGFBP5 transcripts (*p* < 0.01), there were no significant effects of chronic HFD on other RNA markers of cardiac damage in hGH-TG mice (Figure 7).

## 3. Discussion

Using a combination of echocardiography and RNA analysis, hearts from 28-week-old male CD-1 hGH-TG CD-1 mice expressing transgenic hGH without endogenous mGH were compared with age-matched hearts from mGH-WT CD-1 mice expressing endogenous mGH. When assessed at 4 weeks, hGH-TG mice weigh ~42% less than mGH-WT mice, which is consistent with their shorter (20%) length from head to tail and their relatively reduced production of GH from puberty [16]. Similarly, hearts are smaller in hGH-TG mice. The hGH-TG mice weigh ~31% less than age-matched mGH-WT mice maintained on an RCD at 24 weeks [16]. There are signs of GH insufficiency. The hGH-TG mice are small, have decreased IGF-I production, and increased adiposity that is also seen with the GH knockout mouse and GH receptor antagonist (GHA) mouse [16]. The GHA mouse has been described as possessing characteristics of human congenital GHD [16,22]. However, hGH secretion will respond positively to a GH-releasing peptide in hGH-TG mice [16], suggesting they might better be described as an inherently or genetically smaller mouse type.

Multiple heart structural parameters were examined by echocardiography in hGH-TG mice maintained on RCD for 28 weeks. These values were consistent with the smaller size of the hGH-TG mouse, including a 48% lower LV mass and ~36% lower stroke volume. Echocardiographic assessment of LV mass will normally take into account LV, IVS, and LVPW thickness in diastole, which were also independently determined, and all were found to be lower in hGH-TG mouse hearts. The ~48% reduction in cardiac output is also consistent with the smaller hGH-TG mouse and heart, and like the decrease in LV mass, corresponds to the reported effect of GH deficiency in adults without GH replacement therapy [3,23]. Levels of PRL, and by extension, lactogenic signaling via the PRLR, are not consistently associated with the risk of cardiovascular events, even though excess prolactin elevates the risk of endothelial dysfunction and metabolic disorders [14]. Thus, while a possible effect of lactogenic signaling via the ability of hGH to signal through the PRLR cannot be ruled out, the evidence suggests that lower GH levels and, as a result, reduced GHR-related signaling most likely account for the effects on heart structure in hGH-TG mice.

The hGH-TG mouse hearts did, however, show indications of diastolic dysfunction based on mitral pulse wave and tissue Doppler measurements. Specifically, the hGH-TG mice exhibited a higher mean mitral valve (MV) E/A ratio, above 2.0, which is suggestive of impaired LV compliance [24]. By contrast, the average E/A ratio for mGH-WT mouse hearts was in the normal range [24]. In addition, the E/e′ ratio, which is associated with LV filling pressure, was also significantly higher in hGH-TG mice. In humans, this ratio is known to increase as diastolic dysfunction progresses, and a ratio >15 is considered abnormal [25], but an equivalent relationship has not been determined for mice and may not apply given differences in heart rate and heart size between species [26]. An alternative explanation for an E/A ratio >2.0 is supernormal diastolic function. A strong recoil during diastole can generate negative pressure in the ventricle of individuals engaged in physical endurance activity, such as triathletes, causing blood to be pulled from the atrium during early filling, giving rise to a high E-wave and low A-wave, potentially yielding an E/A ratio >2.0 [27,28]. However, assuming the increased MV E/A and E/e′ ratios are indicative of restricted filling of the left ventricle, then this might be explained by LV chamber stiffness. The ratio of E/e′ divided by LV end-diastolic volume (E/e′/LVEDV) has been used as a surrogate method to determine indices of LV chamber stiffness [29,30], and this ratio was significantly greater than 2-fold higher hGH-TG when compared to mGH-WT mouse hearts.

In spite of the elevated MV E/A ratio and a value above 2.0, hGH-TG mice did not display evidence of systolic dysfunction. Percent EF was similar in both hGH-TG and mGH-WT mice with mean values above 50% that, while low, are comparable to values reported by others with anesthetized mice [31]. In addition, even with the additional stress associated with chronic HFD for 24 weeks and some indication of elevated insulin resistance, including impaired glucose clearance and increased liver lipid accumulation, there were no significant adverse effects on EF and FS or further effect on the MV E/A ratio.

The absence of clear systolic dysfunction in hGH-TG mice at 28 weeks in spite of evidence of impaired LV compliance and potential restricted ventricular filling raises the possibility of a compensatory response associated with these hearts. Improvements in diastolic dysfunction in humans have been seen with effective lowering of the heart rate [32]. Specifically, β-blockers can be used to treat diastolic dysfunction by reducing heart rate and increasing the duration of diastole [32,33]. In this context, the heart rate for hGH-TG mice is ~432 bpm at 28 weeks, which is significantly lower than ~528 bpm for mGH-WT mice. Obesity has been linked to an increase in heart rate; however, chronic HFD resulted in a >10% decrease in heart rate to ~380 bpm in hGH-TG mice.

Both the lower heart rate in hGH-TG and the apparent decrease in heart rate in hGH-TG mice with HFD correlate with lower BNP RNA levels. BNP is a member of a family of five natriuretic peptides that also includes ANP, which help regulate blood circulation through effects on blood vessel dilation [34], essentially lowering blood volume, reducing cardiac output, and reducing systemic blood pressure [19]. BNP levels will normally increase due to elevated gene expression and secretion in response to multiple stress-related conditions where there is cardiac dysfunction [19,35], such as increases in LV end-diastolic pressure, atrial pressure, LV hypertrophy, and other conditions detected by echocardiography [35]. Furthermore, BNP levels will normally increase with the degree and progression of these conditions [35,36]. However, low BNP levels, often associated with obesity, have been described in as much as 4–17% of patients with heart failure, abnormal cardiac structure and function, or hemodynamics [37,38], raising the possibility that a natriuretic peptide deficiency may exist, increasing susceptibility to cardiac hypertrophy [37]. Furthermore, BNP as well as ANP production was decreased in obese mice with elevated cardiac ventricle lipid accumulation, regardless of normal or impaired cardiac function [38].

In this context, although lower BNP but not ANP RNA levels were detected in hGH-TG versus mGH-WT mice, the hGH-TG mice have increased total body fat relative to mGH-WT mice, which is associated with some evidence of GH insufficiency [16]. Relative increases in serum low-density lipoprotein, high-density lipoprotein, and total cholesterol were previously observed in hGH-TG mice fed an HFD, although they did not reach significance; however, liver triglyceride accumulation was increased significantly [16]. However, the possibility that lower BNP gene expression is a genetic consequence of transgenesis and potentially affecting the hGH-TG mouse heart cannot be ruled out, given BNP is produced by the atrial and ventricular myocardium during embryonic development with levels downregulated after birth [19].

An increase in IGFBP5 and a decrease in RNF207 RNA levels were identified as markers of cardiac injury related to high blood pressure, based on a whole transcriptome analysis in the mouse [21]. While there was a significant increase in IGFBP5 transcripts, a reduction in RNF207 RNA levels was not observed in hGH-TG mice fed an HFD. There was also no effect on collagen type I and III expression, consistent with a lack of picro-sirius staining and no echocardiographic evidence of non-compensated hypertrophy. More specifically, the weight of the left ventricle is reported to represent the cumulative effect of blood pressure on the heart [39], and no significant effect of chronic HFD for 24 weeks on hGH-TG mouse LV mass was detected. Again, this suggests little or no contribution of hGH lactogenic activity on blood pressure in hGH-TG mice, given that excess PRL is expected to elevate blood pressure in mice [40]. Furthermore, this lack of support for hypertension-related cardiac damage or remodeling may not be surprising given the absence of an effect of HFD on blood pressure reported in inbred C57BL [41] and outbred CD-1 mice [42].

In summary, the hGH-TG mice have multiple characteristics in common with congenital GHD, which now includes smaller heart size and reduced cardiac output. Structural differences between hGH-TG and mGH-WT mouse hearts detected by echocardiography can largely be attributed to GH insufficiency and likely lower GHR as opposed to PRLR-related signaling. However, a contribution of lactogenic activity to increased metabolic stress and insulin resistance in hGH-TG mouse hearts as a result of chronic HFD cannot be ruled out. Similarly, GH levels and/or activities are implicated in the suggested development of impaired LV compliance in the male hGH-TG mouse heart as well as any compensatory effect, including potentially a reduced heart rate and/or lower BNP gene expression, that results in a normal ejection fraction. Although heart size likely reflects the smaller hGH-TG mouse phenotype, GH/IGF-I signaling is linked to apoptosis. Thus, further studies would be required to measure the apoptosis rate of cardiac cells in hGH-TG mice to assess a possible contribution. Finally, given possible differential effects due to the potential for lactogenic as well as somatogenic signaling by hGH, assessment in female hGH-TG mice is warranted. Certainly, further studies using synthetic GHs to potentially counteract the deficits suggested in hGH-TG mice could be pursued to differentiate and/or confirm a role for GH and/or PRL-related signaling.

## 4. Materials and Methods

### 4.1. Mice and Diet

Four-week-old male hGH-TG and mGH-WT CD-1 mice were maintained on a regular chow diet (RCD; fat = 14 kcal%; carbohydrate = 60 kcal%; protein = 26 kcal%) for up to 28 weeks [43]. For diet studies, four-week-old male hGH-TG mice were given RCD for the first 4 weeks and then switched to a high-fat diet (HFD; fat = 60 kcal%; carbohydrate = 20 kcal%; protein = 20 kcal%; per 773.85 g: protein = casein, 200.00 g, and cystine–L 3.00 g; carbohydrate = Lodex 10, 125.00 g, and sucrose, 72.80 g; fiber = Solka Floc, FCC200, 50.00 g; fat = lard, 245.00 g, soybean oil, USP 25.00 g; mineral = S10026B, 50.00 g; vitamin = choline bitartrate, 2.00 g, and vitaminV10001C, 1.00 g; and dye = Blue FD&C #1, Alum. Lake 35–42%, 0.05 g; Research Diets Inc., D12492) for 24 weeks or maintained on an RCD. All mice were housed in an environmentally controlled room maintained on a 12 h light/dark cycle (6 a.m.–6 p.m). Access to food in the form of palatable pellets and tap water was *ad libitum*.

At the end of the study, mouse body weight was obtained, and, after euthanization by cervical dislocation, hearts were weighed and, together with liver samples, fixed in 10% formalin in phosphate-buffered saline for 24 h. Paraffin embedding, transverse sectioning (20 × 5 μm) through the heart to allow both ventricular chambers to be assessed, and staining of nuclei and cytoplasm with hematoxylin and eosin (H&E) or collagen fibers with picro-sirius red were performed by the Histology Services, Imaging Facility, Electron Microscopy Platform at the University of Manitoba, Winnipeg, MB, Canada. Images were captured on a Zeiss Imager M2 Scan (Oberkochen, German).

### 4.2. Glucose Tolerance Test (GTT)

A GTT was performed on 28-week-old adult mice essentially as described previously [44]. In brief, all mice were weighed, fasted for approximately 16 h, and then a GTT was performed using 2 g/kg of intraperitoneal glucose (Sigma, G7258) dissolved in double-distilled water. Blood was collected from a tail vein, and glucose levels (mM) were measured prior to injection (0 min) and then periodically and specifically at 120 min after the glucose injection using a glucose meter (OneTouch Ultra2 Glucose Monitoring System, Lifescan, Inc., Malvern, PA, USA) [45].

### 4.3. Echocardiography

Heart structure and function were assessed using a high-frequency ultrasound with the Vevo 2100 system (Visual Sonics, Toronto, ON, Canada) equipped with a 17.5 MHz transducer. During imaging, the body temperature of the mouse was maintained at 37 °C under mild anesthesia (sedated with 3% isoflurane and 1.0 L/min oxygen and maintained at 1–1.5% isoflurane and 1.0 L/min oxygen) essentially as done previously [46]. Structural and functional cardiac parameters were assessed using three imaging formats: brightness (B)-mode, motion (M)-mode, and Doppler imaging. Cardiac structural parameters assessed included thickness (mm) of the left ventricle anterior and posterior wall (LVAW and LVPW), left ventricle internal dimension and diameter (LVID and LVD), and interventricular septum (IVS) at diastole and systole, as well as left ventricular volume (LV Vol), left ventricular mass (LV Mass), and stroke volume (Stroke Vol). Cardiac functional parameters measured included isovolumic contraction time (IVCT), isovolumic relaxation time (IVRT), left ventricle isovolumic myocardial performance index (LV MPI), left ventricle ejection fraction (EF), fractional shortening (FS), rate at which the atrial and ventricular pressures equilibrate after onset of the E wave (MV E/A), mitral inflow E wave to early diastolic mitral annular tissue velocity (E/e′), ratio of E/e′ divided by left ventricular end-diastolic volume (LV Vol-d), heart rate, and cardiac output (CO). Data were analyzed using the cardiovascular software package from Visual Sonics VEVO 2100 (Version 1.6.0). Measurements were averaged over four cardiac cycles. All images were recorded and analyzed by a trained and blinded research animal echocardiographer.

### 4.4. Real-Time Reverse Transcriptase-Polymerase Chain Reaction (PCR)

Cardiac ventricular tissue was collected and flash-frozen after euthanasia by cervical dislocation before isolation of total RNA. Pituitary RNA was extracted using the RNeasy Plus Mini Kit (Qiagen, Toronto, ON, Canada) with the Qiagen QIAshredder™ 250 Kit. RNA was extracted using the RNeasy Plus Universal Mini Kit (Qiagen, Toronto, ON, Canada). RNA (1 μg) was reverse transcribed using the QuantiTect Reverse Transcription Kit (Qiagen, Toronto, ON, Canada). Quantitative PCR (qPCR) was performed using specific primers for ANP, BNP, COL I, COL III, IGFBP5, RNF207, and hypoxanthine guanine phosphoribosyltransferase (HPRT) gene transcripts, with primer sequences as described (Table 1). The gene expression level in each sample (absolute quantification) was calculated from a standard curve and normalized to mouse HPRT expression as appropriate. Tests were run in duplicate on seven independent samples.

### 4.5. Statistical Analysis

Prism software (10.2.2) was used for statistical analysis. The *t*-test was applied for single comparisons (mouse type or diet), and two-way analysis of variance (ANOVA) with a *post hoc* Tukey test was used for multiple (diet and time or mouse type and diet) group analyses. A z-score was determined for data values, and scores above +3.29 or below −3.29 were considered outliers and excluded from statistical analyses [47]. The final results are expressed as the mean, plus or minus the standard error of the mean, which was either the absolute number or arbitrarily set to 1 or 100%. Mean values were considered significantly different if *p* < 0.05; */^#^ *p* < 0.05, **/^##^ *p* < 0.01, and ***/^###^ *p* < 0.001.

## Figures and Tables

**Table 1:** PCR primers used to detect mouse RNA levels.

RNA	Primer Sequence
Atrial natriuretic peptide (ANP)	5′-GCTTCGGGGGTAGGATTGAC-3′ 5′-CACACCACAAGGGCTTAGGA-3′
B-type natriuretic peptide (BNP)	5′-GCTGGGAGGTCACTCCTATC-3′ 5′-CTTTTGTGAGGCCTTGGTCCTTC-3′
Collagen type I (COL I)	5′-CTGCTCCTCTTAGGGGCCA-3′ 5′-CGTCTCACCATTGGGGACCCT-3′
Collagen type I (COL III)	5′-GGTTTCTTCTCACCCTTCTTC-3′ 5′-GGTTCTGGCTTCCAGACATC-3′
Hypoxanthine guanine phosphoribosyltransferase (HPRT)	5′-CTCATGGACTGATTATGGACAGGAC-3′ 5′-GCAGGTCAGCAAAGAACTTATAGCC-3′
Insulin-like growth factor binding protein 5 (IGFBP5)	5′-CTCGTGAGCTACAAGTGTGG-3′ 5′-TGGGCTATGCACTTGATGCA-3′
Ring finger protein 207 (RNF207)	5′-TGCTGAAGACACCCAGCTGT-3′ 5′-GCAGACCCCGATAGGAATCC-3′

## Data Availability

The original contributions presented in the study are included in the article, further inquiries can be directed to the corresponding author/s.
